# A phase I study of multi-HLA-binding peptides derived from heat shock protein 70/glypican-3 and a novel combination adjuvant of hLAG-3Ig and Poly-ICLC for patients with metastatic gastrointestinal cancers: YNP01 trial

**DOI:** 10.1007/s00262-020-02518-7

**Published:** 2020-03-26

**Authors:** Masao Nakajima, Shoichi Hazama, Koji Tamada, Keiko Udaka, Yasunobu Kouki, Toshinari Uematsu, Hideki Arima, Akira Saito, Shun Doi, Hiroto Matsui, Yoshitaro Shindo, Satoshi Matsukuma, Shinsuke Kanekiyo, Yukio Tokumitsu, Shinobu Tomochika, Michihisa Iida, Shin Yoshida, Yuki Nakagami, Nobuaki Suzuki, Shigeru Takeda, Shigeru Yamamoto, Shigefumi Yoshino, Tomio Ueno, Hiroaki Nagano

**Affiliations:** 1 Department of Gastroenterological, Breast and Endocrine Surgery, Yamaguchi University Graduate School of Medicine, 1-1-1 Minami-Kogushi, Ube, Yamaguchi 755-8505 Japan; 2 Department of Translational Research and Developmental Therapeutics Against Cancer, Yamaguchi University School of Medicine, 1-1-1 Minami-Kogushi, Ube, Yamaguchi 755-8505 Japan; 3 Department of Immunology, Yamaguchi University Graduate School of Medicine, 1-1-1 Minami-Kogushi, Ube, Yamaguchi 755-8505 Japan; 4 Department of Immunology, Kochi Medical School, Nankoku, Kochi 783-8505 Japan; 5 Department of Pharmacy, Yamaguchi University Hospital, 1-1-1 Minami-Kogushi, Ube, Yamaguchi 755-8505 Japan; 6 Department of AI Applied Quantitative Clinical Science, Tokyo Medical University, 6-1-1 Shinjuku, Shinjuku, Tokyo 160-8402 Japan; 7 CYTLIMIC Inc, Shinagawa, Tokyo 141-0021 Japan; 8 Oncology Center, Yamaguchi University Hospital, 1-1-1 Minami-Kogushi, Ube, Yamaguchi 755-8505 Japan; 9 Department of Digestive Surgery, Kawasaki University School of Medicine, 577 Matsushima, Kurashiki, Okayama 701-0192 Japan

**Keywords:** Peptide vaccination therapy, Multi-HLA binding, HLAG-3Ig plus Poly-ICLC, Gastrointestinal cancers

## Abstract

**Background:**

This phase I study aimed to evaluate the safety, peptide-specific immune responses, and anti-tumor effects of a novel vaccination therapy comprising multi-HLA-binding heat shock protein (HSP) 70/glypican-3 (GPC3) peptides and a novel adjuvant combination of hLAG-3Ig and Poly-ICLC against metastatic gastrointestinal cancers.

**Methods:**

HSP70/GPC3 peptides with high binding affinities for three HLA types (A*24:02, A*02:01, and A*02:06) were identified with our peptide prediction system. The peptides were intradermally administered with combined adjuvants on a weekly basis. This study was a phase I dose escalation clinical trial, which was carried out in a three patients’ cohort; in total, 11 patients were enrolled for the recommended dose.

**Results:**

Seventeen patients received this vaccination therapy without dose-limiting toxicity. All treatment-related adverse events were of grades 1 to 2. Peptide-specific CTL induction by HSP70 and GPC3 proteins was observed in 11 (64.7%) and 13 (76.5%) cases, respectively, regardless of the HLA type. Serum tumor marker levels were decreased in 10 cases (58.8%). Immunological analysis using PBMCs indicated that patients receiving dose level 3 presented with significantly reduced T cell immunoglobulin and mucin-domain containing-3 (TIM3)-expressing CD4 + T cells after one course of treatment. PD-1 or TIM3-expressing CD4 + T cells and T cell immunoreceptor with immunoglobulin and ITIM domains (TIGIT)-expressing CD8 + T cells in PBMCs before vaccination were negative predictive factors for survival.

**Conclusions:**

This novel peptide vaccination therapy was safe for patients with metastatic gastrointestinal cancers.

**Electronic supplementary material:**

The online version of this article (10.1007/s00262-020-02518-7) contains supplementary material, which is available to authorized users.

## Introduction

Immunotherapy is currently considered first-line treatment for cancers, owing to the great success of immune checkpoint blockade (ICB). ICB re-invigorates exhausted TAA-specific T cells; therefore, the prevalence of these cells is essential for the ICB efficacy [1, 2]. Therapeutic cancer vaccines comprise antigens and adjuvants. According to numerous early-phase clinical trials, they can induce TAA-specific CTL responses and have certain therapeutic efficacy among some patients with advanced gastrointestinal (GI) cancers [3–5]. However, nearly all late-phase clinical trials with therapeutic cancer vaccines have demonstrated relatively limited efficacy [6–8].

The inadequate clinical efficacy of most therapeutic cancer vaccines may be attributed to various obstacles, including TAA-specific T cell exhaustion and HLA restriction. TAA-specific T cells are activated in lymphoid organs. Nevertheless, their cytotoxicity and persistence are strongly suppressed via multiple inhibitory co-signals in the tumor microenvironment; this state is called exhaustion [9]. To overcome immune exhaustion, we focused on adjuvant optimization. We have previously reported that combinatorial administration of poly(I:C) and LAG-3-Ig adjuvants synergistically stimulated tumor-specific T cell responses, prevented T cell exhaustion, and enhanced the therapeutic efficacy of cancer vaccines in a preclinical mouse model [10]. Therefore, this adjuvant combination potentially exerts therapeutic effects among patients with GI cancers. To date, however, no clinical studies have been performed with these adjuvant combinations. The current study is the first to evaluate this adjuvant combination.

HLA-A*02:01 and 24:02 have been the focus of cancer vaccination therapy worldwide [11, 12]. HLA-A*02:01 is expressed in Japanese (~ 40%) and other ethnic populations and in ~ 50% of Caucasians, while HLA-A*24:02 is expressed in Japanese, other Asian, and Latino populations (~ 60%) [13]. Therefore, restricting the cancer peptide to one HLA allele deters the application of vaccine therapies to larger populations. To overcome HLA restriction, we previously generated multi-HLA-binding peptides from heat shock protein (HSP) 70, an immunogenic tumor antigen [14, 15], with a novel peptide prediction system developed by the NEC Corporation, and we confirmed their capacity for CTL induction and CTL cytotoxicity against tumor cells expressing HSP70 proteins [16].

Recently, targeting multiple TAAs as cancer vaccines has been reported to be essential for preventing immune evasion in heterogeneous cancers [17, 18]. Therefore, we explored a novel vaccination therapy using a combination of HSP70-derived multi-HLA-binding peptide with another peptide complementary to HSP70 protein for application in various cancers. We focused on carcinoembryonic antigen glypican-3 (GPC3) as a candidate partner protein for HSP70. GPC3 is an ideal target for immunotherapy in HCC because it has been reported to be specifically overexpressed in it (72–81%) and to be correlated with a poor prognosis [19, 20]. It was also reported that GPC3 is overexpressed in numerous tumors [21]. Several clinical trials were conducted using GPC3-targeted peptide vaccination-, antibody-, and chimeric antigen receptor-regenerated T cell immunotherapies and established their efficacy in patients with solid tumors [12, 22, 23]. Therefore, we decided to explore a multi-HLA-binding peptide from GPC3 protein as a candidate partner peptide for HSP70 for our vaccination therapy.

Here, we report a phase I study of cancer vaccination using a combination of two multi-HLA-binding peptides, HSP70 and GPC3, and the novel immune adjuvants combination of Poly-ICLC and hLAG-3Ig for patients with metastatic GI cancers. This study aimed to evaluate the safety, feasibility, immunological response, and clinical efficacy of this novel vaccination therapy.

## Materials and methods

### Evaluation of HSP70 and GPC3 expression

HSP70 and GPC3 proteins are upregulated in HCC [15, 19]. To determine which types of cancer express HSP70 and GPC3, IHC analysis was performed using large tissue sections. Pancreatic ductal adenocarcinoma (PDAC), liver, esophageal, gastric, colorectal, and breast cancers were evaluated (Table 1). Antihuman HSP70 (clone C92F3A-5, 1 μg mL^−1^; Abcam, Cambridge, UK) and antihuman GPC-3 (clone 1G12, 1.67 μg mL^−1^; BioMosaics, Burlington, VT, USA) and antibodies were used for IHC analysis. IHC results for HSP70 and GPC3 were rated negative, weakly positive, positive, or strongly positive. Cases wherein scores ≥ weakly positive were defined as expression-positive cases. IHC staining was analyzed by two certified Japanese pathologists blinded to the clinicopathological parameters.

**Table 1:** Expression of HSP70 and GPC3 on various cancers

Types of tumor	No. of samples	Positivity of antigen (%)		
		HSP70	GPC3	HSP70 + GPC3
Liver cancer				
HCC	112	71	71	87
CCC	10	100	30	100
CHC	10	100	90	100
Total	132	75	70	89
PDAC	21	81	14	86
ESCC	15	100	40	100
Gastric cancer	22	82	27	86
CRC	24	75	4	79

*CCC* cholangio cellular carcinoma, *CHC* combined hepatocellular carcinoma

### Identification of multi-HLA-binding HSP70 and GPC3 peptide

The HSP70 peptide was identified as previously reported [16]. This identification method was used to determine the GPC3-derived epitope immunogenic peptide exerting cytotoxicity against multi-HLA-A types (HLA-A*24:02, 02:01, and 02:06). In brief, the prediction system of HLA- peptide binding score developed by NEC Corporation was used to select candidate peptides derived from GPC3. The candidate GPC-derived epitope peptides were shortlisted by assessing their binding capacities to multi-HLA types. Then, peptide binding to each HLA-A type was determined via an acid stripping and reconstitution assay as previously described by Zeh et al., with minor modifications [24]. Finally, a single peptide was identified among those whose immune induction capacities were confirmed via an in vitro IFN-γ ELISPOT assay on PBMCs from patients (Table 2).

**Table 2:** Selection of GPC3 peptide by binding assay and ELISPOT assay

	Peptide (Amino acid sequence)		
	GPC-1* (MVNELFDSL)	GPC-2 (LFDSLFPVI)	GPC-3 (SLQVTRIFL)
HLA allele	Binding assay (Log Kd)		
24:02	− 4.7	− 7.43	− 5.34
02:01	− 5.31	− 5.07	− 6.21
02:06	− 6.14	> − 3	− 5.21
	ELISPOT assay		
24:02 (except 02:01, 02:06)	2/3	0/3	0/3
02:01 (except 24:02, 02:06)	1/2	1/2	2/2
Other combination	1/2	0/2	2/2
Total	4/7	3/6	4/7

### Peptide production

The 9-mer peptides derived from HSP70 (YGAAVQAAI) and GPC3 (MVNELFDSL) were generated for the clinical trial in accordance with Good Manufacturing Practice (GMP) by Sekisui Medical Co. Ltd. (Tokyo, Japan) and purified by HPLC to a final purity of > 99%.

### Study design and endpoints

This study was a phase I dose escalation clinical trial on patients with metastatic GI cancers and was designated the Yamaguchi University and NEC Peptide (YNP) 01 trial. The primary objectives of this study were to evaluate the safety and establish recommended doses (RD) of HSP70, GPC3, hLAG-3Ig, and Poly-ICLC. Our secondary objectives were to evaluate peptide-specific immune responses and anti-tumor effects, OS, PFS, response rate (RR), and disease control rate (DCR). DCR is defined as the proportion of patients who have the best response rating of complete response (CR), partial response (PR) or stable disease (SD) according to the RECIST guidelines (v. 1.1). Exploratory objectives included evaluation of the surface markers on PBMCs before, during, and after treatment. The study protocol was approved by the Institutional Ethics Review Boards of Yamaguchi University (No. H27-108), conducted in accordance with the Helsinki Declaration on experimentation on human subjects, and deposited in the UMIN Clinical Trials Registry as UMIN000020440.

### Inclusion and exclusion criteria

Patients eligible for enrollment had histologically confirmed metastatic GI cancer, failed to respond to, or could not tolerate, standard therapy, and presented with the HLA-A*24:02, HLA-A*02:01, or HLA-A*02:06 alleles. The patients were monitored for ≥ 2 weeks from the end of prior treatment to the initiation of the new vaccine regimen to allow patients to recover completely from ≥ grade 3 adverse events in accordance with the Common Terminology Criteria for Adverse Events v. 4.0 (CTCAE). Patients were required to have an Eastern Cooperative Oncology Group (ECOG) performance status (PS) of 0 or 1, be > 20 years old, with a life expectancy of ≥ 3 months. Other prerequisites included adequate bone marrow, renal, and liver function (Hb ≥ 8.0 g dL^−l^, leukocyte count ≥ 2000 mm^−3^, lymphocyte proportion ≥ 15%, platelet count ≥ 75,000 mm^−3^, serum creatinine ≤ 2.5 × the institutional upper limit of normal (ULN), total bilirubin ≤ 2.0 mg dL^−l^, and transaminase ≤ 2.5 × the institutional ULN). Eligible patients also had ≥ 1 measurable lesion.

Patient exclusion criteria included pregnancy, a second primary tumor, severe ischemic heart disease, inadequately controlled organ dysfunction, brain metastasis, past history of pulmonary fibrosis or interstitial pneumonia clearly visible on chest radiography, active infectious disease, steroid-dependent autoimmune diseases, or prior or the current treatment with HSP70, GPC3, hLAG-3Ig, or Poly-ICLC. Written informed consent was obtained from patients at enrollment.

### Treatment schedule

Figure S1A shows the dose escalation and cohort assignment of the present study. Briefly, dose escalation was carried out in a three-patient cohort using 1 or 2 mg HSP70 and GPC3 combined with 250 or 1000 μg hLAG-3Ig/IMP321 (Immutep S.A., Châtenay Malabry, France) and 1.4 mg Poly-ICLC/Hiltonol (Oncovir, Inc., Washington, DC, USA). Levels 1/2 and level 3 dosages of IMP321 were set as 250 μg/injection and 1000 μg/injection, respectively. Previously, 250 μg/injection of IMP321 into one dermal site was reported as safe and effective for inducing CTLs. As treatments were administered at four sites, the IMP321 dose was escalated by 4 × (1000 mg/body) [25, 26]. According to previous studies, a fixed Hiltonol dosage of 1.4 mg/body was considered appropriate for vaccination therapy. As this agent influences systemic inflammation, a fourfold increase in injection dose might have been highly toxic [27]. The peptide/adjuvant mixtures were intradermally injected into four sites (bilateral thigh and axilla regions) on days 1, 8, 15, and 22 of each of the two 28-d treatment courses. From the third treatment course onwards, the vaccinations were administered biweekly. By the fifth treatment course, the treatments were reduced to once every 4 weeks. Vaccination was continued after disease progression upon the requests of the patient and/or a primary physician.

Dose-limiting toxicities (DLTs) were evaluated during the first treatment cycle (28 d). The major DLTs were defined as CTCAE v. 4.0 ≥ grade 4 hematological toxicity or ≥ grade 3 non-hematological toxicity. Exceptions were ≥ grade 3 injection site reaction, nausea, and vomiting.

### Clinical evaluations

Baseline evaluations included a medical history, physical examination, vital signs, ECOG performance status, height, weight, chest X-ray, electrocardiography, routine blood analysis (hematology and chemistry), cancer type-related tumor marker measurements, and computerized tomography (CT) or magnetic resonance imaging (MRI). All aforementioned assessments were performed within 21 d before treatment onset. Physical examination, hematology, and biochemical analyses were repeated on day 1 of each treatment cycle. Tumor biopsies were performed before and after treatment wherever possible. Tumor assessments (CT, MRI, and serum tumor markers) were repeated every 4 weeks after initial treatment. The RECIST guidelines (v. 1.1) as well as the immune-related response criteria (ir-RC) were used to define all responses. Signs of toxicity were assessed in accordance with CTCAE (v. 4.0). Associations between adverse events and treatments were classified as definite, probable, possible, not likely, or not related. Definite, probable, and possible adverse events were certified as indicators of treatment-related toxicity. Blood samples were drawn before each treatment course, and PBMCs were isolated and preserved in liquid nitrogen until subsequent analysis.

### Immunomonitoring

PBMCs were used in the IFN-*γ* ELISPOT assays and surface marker expression assessments. The surface markers included exhaustion markers (PD-1, T cell immunoglobulin and mucin-domain containing-3; TIM-3, LAG-3, and T-cell immunoreceptor with immunoglobulin and ITIM domains; TIGIT) and immunosuppressive cells (Treg cells identified as CD4 + CD25 + CD45RA- cells and MDSCs identified as CD11b + CD33 + HLA-DR- cells). Monoclonal antibodies used herein were purchased from BioLegend (San Diego, CA, USA), Beckton Dickinson (Franklin Lakes, NJ, USA), Beckman Coulter (Brea, CA, USA), or Miltenyi Biotec (Auburn, CA, USA), unless otherwise specified, comprising anti-CD8 (clone HIT8a), anti-CD4 (clone VIT4), anti-PD-1 (clone EH12.2H7), anti-LAG-3 (clone 7H2C65), anti-TIM-3 (clone F38-2E2), anti-TIGIT (clone A15153G), anti-CD62L (clone DREG-56), anti-CD45RA (clone HI100), anti-CD25 (clone B1.49.9), anti-CD11b (clone Bear1), anti-CD33 (clone WM53), and anti-HLA-DR (clone Immu-357) antibodies. Flow cytometry was performed using a MACSQuant Analyzer 10 (Miltenyi Biotec, Auburn, CA, USA), and the output was analyzed using FlowJo (FlowJo LLC, Ashland, OR, USA).

### Measurement of peptide-specific IFN-*γ* response

Antigen-specific T cell responses were estimated using ELISPOT assays following in vitro sensitization, as previously described [28, 29]. In brief, PBMCs were derived from patients before and every 4 weeks after the administration of vaccinations. PBMCs were thawed simultaneously, and 1 × 10^6^ cells/well were incubated in medium with peptide stimulation (final concentration of 10 μg mL^−1^) for 24 h, performed twice on days 1 and 8. A total of 20 IU μL^−1^ of recombinant IL-2 (Novartis) was supplemented on days 2, 5, 9, and 13. On day 15, the cultured lymphocytes were subjected to an ELISPOT assay after negative selection of CD8 + T cells, using magnetic beads (Miltenyi Biotec) and after incubation for 18–20 h in an incubator with peptide-pulsed stimulator cells. The number of peptide-specific spots was determined by subtracting the number of spots in the control well from that in a well containing vaccinated peptide-pulsed stimulator cells. Antigen-specific T cell responses were of grades −, +, 2 + , 3 + , 4 + , or 5 + and based on a previously reported modified algorithm [28, 29] (Fig. S2). The sensitivity of the ELISPOT assay was estimated to be average in accordance with the ELISPOT panel of the Cancer Immunotherapy Consortium. Successful CTL induction was defined by an increase in the grade of peptide-specific spots within three vaccination cycles compared to that in the pre-treatment cycle. Epitope peptides HIV-A*24:02 (RYLRDQQLL), HIV-A*02:01 (SLYNTVATL), and HIV-A*02:06 (ATLEEMMTA) were considered negative controls [30].

### Statistical analysis

Student’s *t* test was used to evaluate peptide-specific immune responses. OS was analyzed using the Kaplan–Meier method. Survival was measured in days from enrollment to death caused by the disease. The correlation between overall survival and surface marker expression in PBMCs before vaccination was analyzed using a Gehan–Wilcoxon test. Correlations among four factors, including antigen expression, antigen-specific T cell induction, reduction in TIM3 + CD4 + T cells, and clinical outcomes, were analyzed using Pearson’s correlation analysis. All statistical analyses were performed in SPSS Statistics v. 17.0 (IBM Corp., Armonk, NY, USA), and *P* < 0.05 was considered statistically significant.

## Results

### Expression of HSP70 and GPC3 proteins in various cancers

**Fig. 1:**
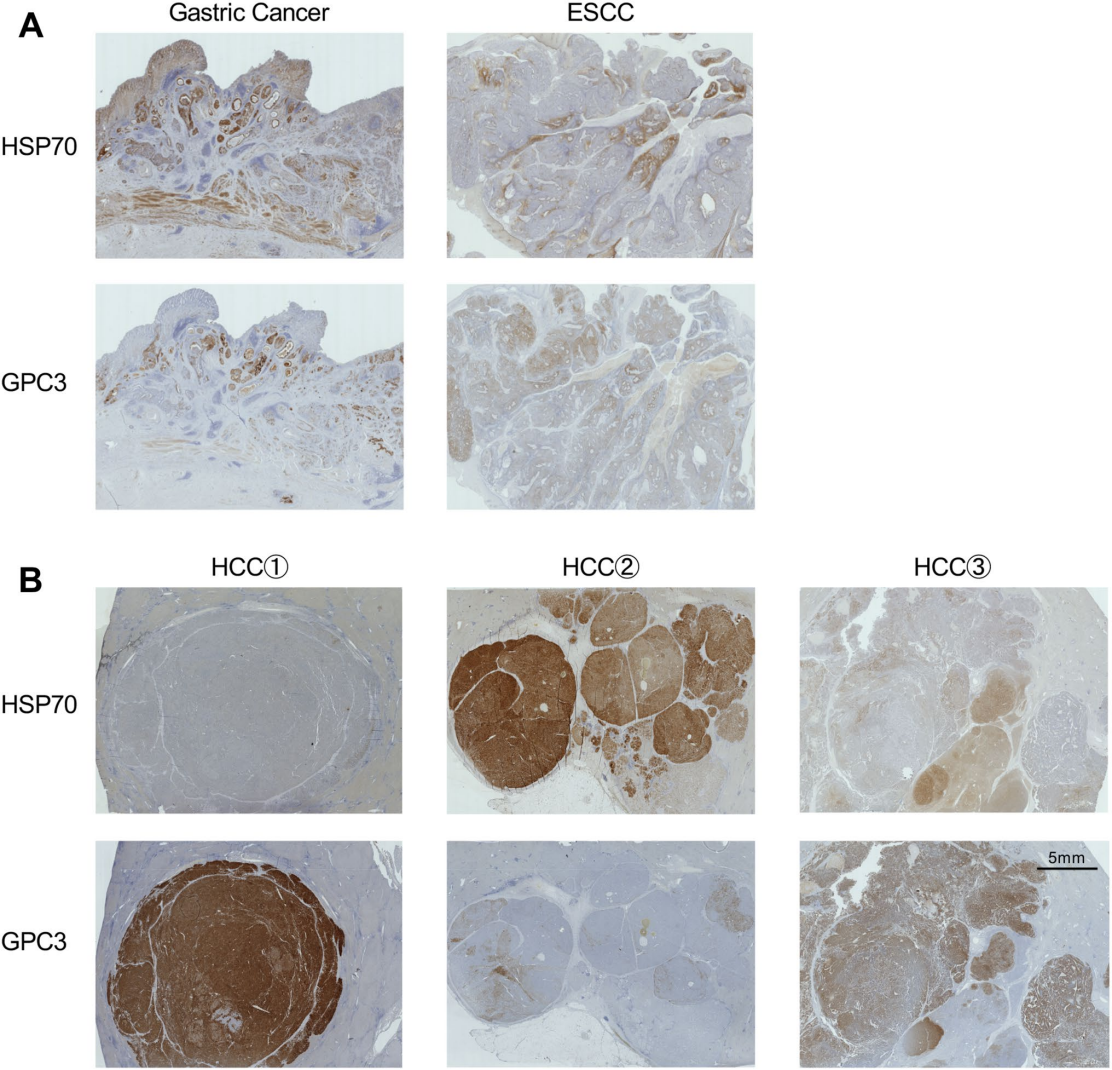
Expression of HSP70 and GPC3 in various gastrointestinal cancers. **a** Expression of HSP70/GPC3 in gastric cancer, esophageal squamous cell carcinoma, and breast cancer. **b** Various expression patterns of HSP70 and GPC3 in hepatocellular carcinoma

The expression profiles of HSP70 and GPC3 were evaluated in various GI cancers. Figure 1 shows representative IHC staining for HSP70 and GPC3. Table 1 shows 214 samples of HCC, PDAC, esophageal squamous cell carcinoma (ESCC), gastric cancer, colorectal cancer (CRC). HSP70 was upregulated (> 70%) in nearly all cancers. However, GPC3 was expressed primarily in HCC (~ 70%) and ESCC (~ 40%). In certain cases, HSP70 and GPC3 expression levels were complementary within the same tumor (Fig. 1). As a result, the combined expression levels of HSP70 and GPC3 were greater than those of either protein alone in HCC, PDAC, gastric cancer, and CRC. Therefore, administration of both peptides derived from HSP70 and GPC3 is potentially applicable to patients with the aforementioned cancers and address the heterogeneity of antigen expression in cancer cells.

### Selection of multi-HLA reactive peptides

To overcome HLA restriction in vaccination therapy, we developed multi-HLA (HLA-A*24:02, 02:01, and 02:06)-binding peptides derived from HSP70 and GPC3, which are potentially applicable and efficacious in various Asian and other ethnic patients. We selected eight candidate peptides from GPC3 protein predicted to have high predictive binding affinities for each HLA-A*24:02, 02:01, and 02:06 (Table S1). Of these, three with relatively higher affinities for all three HLA-As were selected via a in vitro binding assay (Table S1, bold font). Finally, we selected GPC-1 (MVNELFDSL) as an ideal peptide for vaccination therapy because it induced IFN-*γ*-producing CTLs in response to all three HLA types in accordance with an ELISPOT assay (Table 2).

### Patient characteristics

Between January 2016 and September 2017, 23 patients were enrolled in our trial. Six of them were ineligible because of an HLA mismatch. Seventeen HLA-A*24:02-, 02:01-, or 02:06-matched patients (six with CRC, five with EC, four with HCC, and one each with PDAC and Gastric cancer) comprised the study population and received vaccination therapy (73% of the enrolled populations). Patient characteristics and therapeutic evaluations are summarized in Table 3.

**Table 3:** Patients characteristics and therapeutic evaluation

Level	Age	Sex	PS	HLA	Type of tumor	Site of recurrence	Pre-treatment against recurrence	Number of vaccine	CTL induction		Antigen expression		RECIST	OS (m)	PFS (m)
											Primary/metastatic lesion				
									HSP70	GPC3	HSP70	GPC3			
1–1	60	M	0	A*24:02	ESCC	Perit	RT, CT	15	+	+	3 +/3+	1 +/1+	SD	10.5	3
1–2	63	F	0	A*24:02	ESCC	LN	Ct	8	+	–	2 +/3+	−/−	PD	2.4	1
1–3	47	F	0	A*24:02	PDAC	Liver	CT	8	–	+	2 +/2+	−/−	PD	2.8	1
2–1	78	F	1	A*02:06	ESCC	LN	RT, CT	10	+	+	3 +/3+	−/−	SD	6.5	3
2–2	60	M	0	A*02:01	HCC	Lung	CT	39	–	–	2 +/3+	3 +/2+	SD	34 A	34
2–3	78	F	0	A*24:02	HCC	Liver, Bone, LN	RT, CT	13	–	+	1 +/3+	+2/−	PD	6.3	2
3–1	66	M	0	A*24:02	CRC	Liver	RT, CT	13	+	+	ND/3+	ND/−	PD	22.7	2
3–2	66	M	0	A*02:06	CRC	Lung, Pelvis	RT, CT	11	+	–	2 +/ND	−/ND	PD	5.3	2
				A*24:02					+	+					
3–3	62	F	0	A*02:01	CRC	Liver, Lung	CT	10	–	+	1 +/2+	−/−	PD	20.4	2
3–4	63	M	0	A*24:02	HCC	Lung	CT	9	+	+	2 +/ND	3 +/ND	PD	29 A	1
3–5	71	M	0	A*02:01	CRC	Lung, LN	CT	9	+	+	-/3+	1 +/1+	PD	9.6	2
3–6	74	M	0	A*24:02	GC	Liver, LN	CT	13	+	+	3 +/3+	−/−	SD	10.5	3
3–7	65	M	0	A*02:06	HCC	Lung, Bone	RT	7	+	–	ND	ND	SD	23 A	2
				A*24:02					+	–					
3–8	41	F	0	A*02:01	CRC	Lung, Bone, Perit	CT	4	–	–	3 +/3+	−/−	PD	19.6	1
3–9	58	M	0	A*02:06	ESCC	Bone	RT, CT	8	+	+	ND	ND	PD	2.3	1
				A*24:02					+	+					
3–10	69	M	1	A*02:06	ESCC	LN	RT, CT	9	–	+	3 +/ND	−/ND	PD	10.7	2
				A*02:01					–	+					
3–11	64	F	0	A*24:02	CRC	Lung, LN	CT	9	+	+	1 +/2+	−/−	PD	6.3	2

*LN* lymph node, *Perit* peritoneum, *A* still alive, *ND* not done, *OS* overall survival, *PFS* progression-free survival, *GC* gastric cancer, *RT* radiotherapy, *CT* chemotherapy

### Dose escalation and safety

Vaccines were well tolerated, and there were no dose-limiting toxicities during dose escalation. In addition to the three scheduled cases, eight were added; in total, 11 patients were enrolled at the recommended doses of 2.0 mg HSP70 and GPC3 together with 1.4 mg Poly-ICLC and 1.0 mg hLAG-3Ig (Fig. S1A). There were no severe adverse events associated with vaccination therapy in any of the 17 patients at any dose (Table S3). Adverse events associated with vaccination therapy were grade 1 injection site reactions (5/17, 29.4%) and grade 1 edema of the extremities (2/17, 11.7%). As a result, level 3 dosage was considered appropriate for the clinical study.

### Peptide-specific immune responses

Peptide-specific CTL induction by HSP70 and GPC3 proteins was observed in 11 (64.7%) and 13 (76.5%) cases, respectively. Specifically, HSP70-specific CTL inductions were observed in two of three patients at level 1, one of the three patients at level 2, and 8 of 11 patients at level 3 dosage. GPC3-specific CTL inductions were observed in two of three patients each at level 1 and 2, and 9 of 11 patients at level 3. HSP70-specific CTL induction was observed in 9 of 11 patients in HLA-A*24:02, one of the five patients in HLA-A*02:01, and four of five patients in HLA-A*02:06. GPC3-specific CTL induction was observed in 9 of 11 patients in HLA-A*24:02 and three of five patients each in HLA-A*02:01 and HLA-A*02:06. Details of CTL induction are summarized in Table S2. Early and strong CTL induction was defined as *a* > = 3 + grade of peptide-specific spots after one course of vaccination therapy. Early, strong HSP70-specific and GPC3-specific CTL induction was observed in 54.5% and 45% of the patients at level 3, respectively. In contrast, this response was observed in only one patient at levels 1 and 2 (*P* < 0.05) (Table S2).

### Clinical evaluation and overall survival

**Fig. 2:**
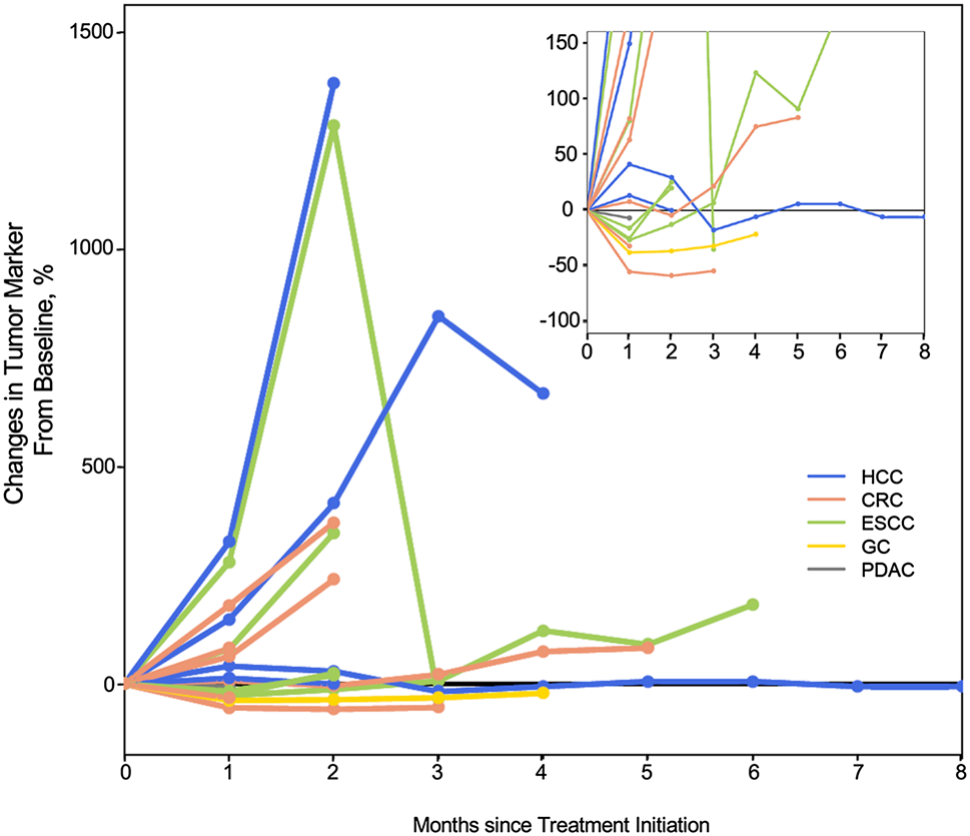
Changes in serum tumor marker levels relative to those under pre-treatment conditions. The tumor markers quantified herein were SCC for esophageal squamous cell carcinoma, CA19-9 for pancreatic ductal adenocarcinoma and colorectal cancer, AFP for hepatocellular carcinoma, and CEA for colorectal cancer and gastric cancer

IHC analysis of the qualified specimens revealed HSP70 expression in 85% of all primary and 100% of all metastatic lesions. However, GPC3 expression was observed in 38% of all primary and 25% of all metastatic lesions. In detail, GPC3 was expressed in 100%, 25%, and 25% of all primary lesions and 50%, 33%, and 25% of all metastatic lesions in HCC, ESCC, and CRC, respectively. In all cases, tumor cells expressed either HSP70 or GPC3 in both primary and metastatic lesions. Table 3 and Fig. S1B show the therapeutic outcomes of vaccination therapy. In one case, lung metastasis in HCC was stabilized for > 2 years. The condition of patients remained stable for 2–34 months, and the DCR was 29%. Unfortunately, in this treatment cohort of 17 patients, there were no patients whose tumor shrank after pseudo-progression. Figure 2 shows the changes in tumor marker levels. Reductions in the levels of serum tumor markers were observed in 10 cases (58.8%). Patients with cancers positive for both antigen proteins, i.e., HSP70 and GPC3, tended to present a better overall survival (*P* = 0.08), and tumor markers were reduced more often in patients with peptide-specific CTL induction for at least one of the two peptides (*P* = 0.031) (Table S4).

### Overall survival and surface marker expression in PBMCs before vaccination

**Fig. 3:**
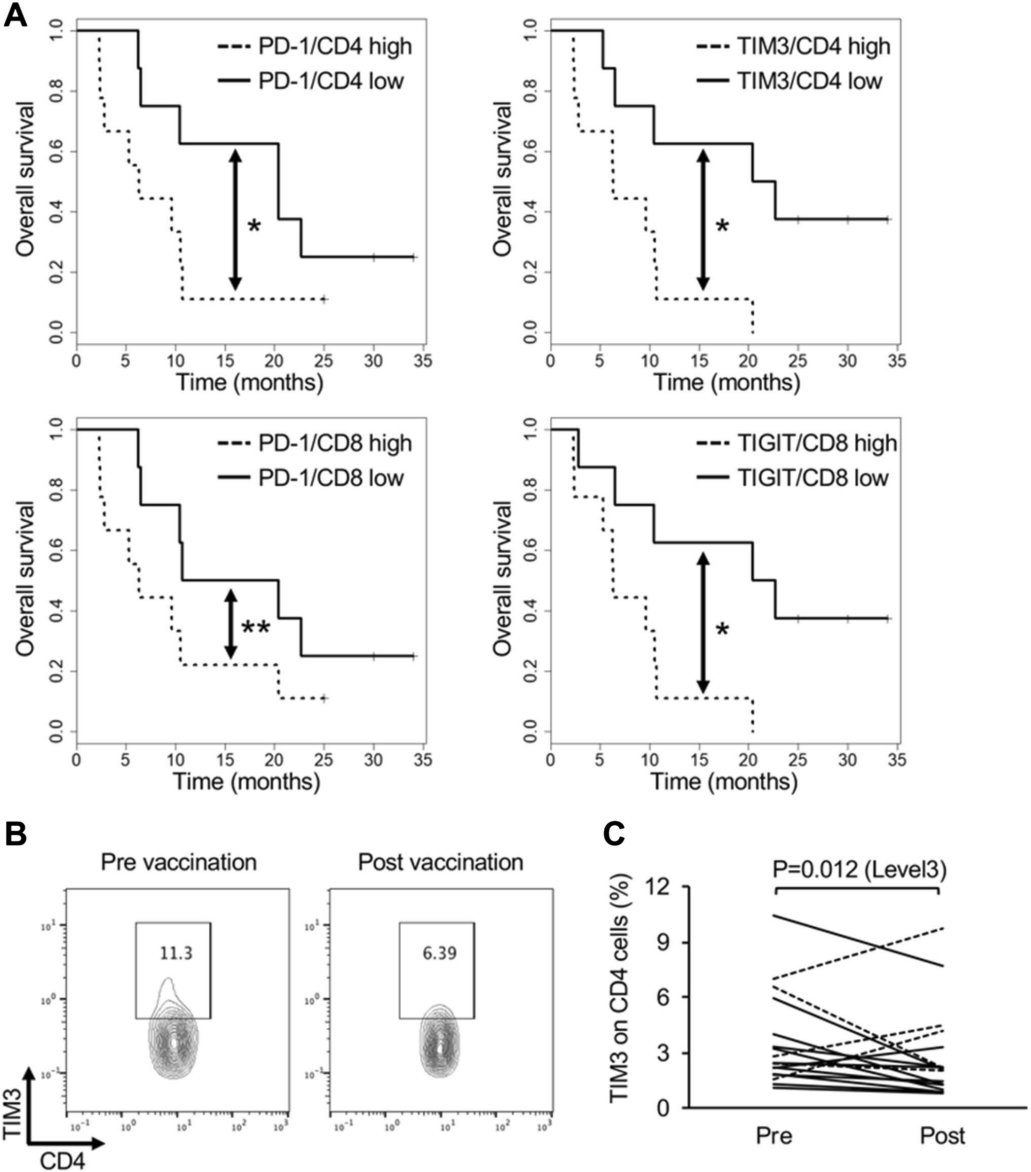
Immunological analysis using PBMCs. **a** Kaplan–Meier curves for overall survival according to pre-treatment PD-1, TIM3 expression levels in CD4 + T cells and PD-1, TIGIT expression levels in CD8 + T cells. **b** and **c** Relative differences in TIM3 expression levels in CD4 + T cells before and after one course of vaccination therapy. Flow cytometry data (**b**) and quantitative analysis (**c**). (*P < 0.05, **P < 0.1)

Suppressive immune cell markers in PBMCs before vaccination were measured, and the associations between these markers expression and OS were assessed. Patients were divided into high (> median) and low (< median) groups on the basis of median values of the proportions of surface marker expression. OS was then compared between pairs of groups (Fig. 3a). There was a significant association between PD-1 expression on the CD4 + T cells, TIM3 expression on the CD4 + T cells, and TIGIT expression on the CD8 + T cells and the OS (*P* = 0.039, 0.025 and 0.032, respectively). Moreover, there was a tendency of association between PD-1 expression on CD8 + T cells and the OS (*P* = 0.058).

### Changes in surface marker expression in PBMCs before and after vaccination therapy

To determine whether vaccination therapy re-invigorated exhausted T cells and improved the systemic immunosuppressive microenvironment, we analyzed surface marker expression in PBMCs before and after one course of treatment. In patients receiving level 3 doses, the proportion of TIM3 + cells in the CD4 + T cells significantly decreased (*P* = 0.012) after one course of treatment in comparison with the pre-treatment condition (Fig. 3b, c). No significant changes were observed in the other cell surface markers before and after one course of treatment (Fig. S3).

## Discussion

In this phase I dose escalation study, HSP70 and GPC3 peptides were combined with Poly-ICLC and hLAG-3Ig adjuvants and administered to patients with metastatic GI cancers. Our novel multi-HLA-binding peptides from HSP70 and GPC3 proteins were applicable to various patients with various types of GI cancers. Our vaccination therapy was safe and strongly induced peptide-specific CTL, especially at recommended dose level 3.

Our study suggested that HSP70 and GPC3 are immunogenic eligible candidate proteins for peptide vaccination therapy, and peptides derived from these proteins can be administered and induce peptide-specific CTLs for patients with various GI cancers and the three common types of HLA alleles. It has been difficult to apply previous vaccination therapies to larger populations, considering that almost all peptides used previously are restricted to one HLA allele [4, 5, 31]. In contrast, our vaccination therapy used novel peptides which can bind to multi-HLA types including HLA-A*24:02, 02:01, and 02:06. Theoretically, this vaccine is suitable for ~ 85% of all Japanese and Latino populations and 60% of all Asian and Caucasian populations, and in fact, 73% of all patients were enrolled herein [13]. Moreover, immunohistochemical examination revealed that > 80% of the cancers tested expressed either HSP70 or GPC3. IHC analysis of the specimens of enrolled patients revealed that all specimens expressed either or both these proteins irrespective of whether lesions were primary or metastatic. In terms of CTL induction, to discover optimized shared-antigen peptides which escape self-tolerance and are immunogenic, we explored cryptic peptides using the peptide prediction system developed by NEC Corporation. Cryptic peptides have intermediate binding affinity toward MHC; therefore, clonal T cell deletion does not occur or only occurs rarely, thus retaining a large TCR repertoire (Menez-Jamet J, et al. Optimized tumor cryptic peptides: the basis for universal neo-antigen-like tumor vaccines. Ann Transl Med 2016;4:266). In this trial, the induction of peptide-specific CTLs was achieved in approximately 70% of patients. Nevertheless, durable anticancer effects were not observed. This may, in part, imply the necessity of CTLs with higher-avidity TCRs against cancer antigens. From the viewpoint of HLA type, GPC3 peptide effectively induced CTLs, regardless of the HLA type (60–90%); on the other hand, the induction rate of HSP70 peptide in patients with HLA-A*02:01 was slightly lower (20%) than that in patients with HLA-A *24:02 and *02:06 (about 80%). Collectively, although our novel vaccination therapy could be applicable to a wide range of patients with various GI cancers and the three common types of HLA alleles, further refinement of our peptide prediction system is warranted.

Kano et al. reported that a combination of poly(I:C) and LAG-3-Ig adjuvants orchestrated multiple, non-overlapping immunostimulatory mechanisms. This process accounted for the profound synergy of therapeutic effects observed in a preclinical mouse model administered the anti-tumor vaccine [10]. In the present study, we confirmed the orchestration of these clinical effects by evaluating peptide-specific CTL induction and surface marker expression in patient PBMCs. Approximately 70% of the patients in this trial showed specific CTL induction to each of the peptides. This finding is superior to those of previous reports [3–5, 32]. Additionally, patients receiving dose level 3 (1.0 mg hLAG-3Ig + 1.4 mg Poly-ICLC/injection) had significantly faster and stronger peptide-specific CTL induction than those at dose levels 1 and 2. The importance of CD4 + T cells and T cell exhaustion in vaccination therapy was recently reported [33–36]. T cell immunoglobulin and mucin-domain containing-3 (Tim-3) is a type I transmembrane protein that was originally discovered as a novel cell surface molecule to mark IFN-*γ*-producing Th1 cells. Recent reports have demonstrated that ligation of Tim-3 ligand and Tim-3 expressed on CD4 + T cells inhibits TCR signaling in T cells and that Tim-3-expressing Foxp3 + regulatory T cells that have enhanced regulatory function play a key role in preventing effective CTL induction and T cell tolerance (Das M, et al. Tim-3 and its role in regulating anti-tumor immunity. Immunol Rev 2017;276:97–111). In the present study, the proportion of TIM-3 expression on CD4 + T cells was significantly decreased after one course of treatment in patients at dose level 3. Considering the foregoing results, we concluded that dose level 3 was the RD and that a high dose of our combined adjuvants may contribute to effective induction of CTLs, possibly by reducing Tim-3 expression on CD4 + T cells or decreasing Tim-3 + Treg cells. This theory was corroborated by previous studies, reporting that only higher doses of IMP321 induced an immune response [25, 37]. Further in vitro and in vivo studies are required.

Although our vaccination therapy could not provide a long-lasting therapeutic effect, we found that patients with low PD-1 or TIM3 expression on CD4 + T cells and TIGIT or PD-1 expression on CD8 + T cells before vaccination therapy had comparatively better OS. Furthermore, patients with cancers positive for both antigen proteins, i.e., HSP70 and GPC3, tended to present a better overall survival (*P* = 0.08) and that tumor markers were reduced more often in patients with peptide-specific CTL induction for at least one of the two peptides (*P* = 0.031). However, other immunological markers were not associated with one another (data not shown). These parameters could be prognostic markers for patients with metastatic GI cancers treated with our vaccination therapy. Previous reports have demonstrated that by re-invigorating exhausted T cells, vaccination sensitivity could be induced, leading to long-lasting therapeutic efficacy against GI cancers [38–40]. Therefore, combinatorial therapy including vaccination therapy and immune checkpoint blockade to re-invigorate exhausted T cells may overcome the limited therapeutic efficacy of our vaccination therapy [41].

Our study is limited by small cohort size of patients with specific cancers, thus deterring the confirmation of the efficacy of our vaccination therapy is warranted. Therefore, we have initiated a novel clinical trial with perioperative vaccination therapy using this vaccine for patients with resectable HCC (No. UMIN000029991).

In conclusion, our novel cancer vaccination therapy comprising two peptides that bind multi-HLAs with Poly-ICLC and hLAG-3Ig adjuvants achieved highly effective induction of antigen-specific CTLs in patients with metastatic GI cancers. Although our vaccination therapy could not demonstrate long-lasting therapeutic efficacy against metastatic GI cancers, we believe that our novel methods, particularly the protocol to generate multiple HLA-binding peptides and the novel combinations of adjuvants with the potential to prevent T cell exhaustion, might be applicable to other vaccination therapies and may facilitate the development of new immunotherapeutic strategies.

### Electronic supplementary material

Below is the link to the electronic supplementary material.

Supplementary material 1 (PDF 57 kb)

Supplementary material 2 (DOCX 19 kb)

Supplementary material 3 (PDF 757 kb)

## Data Availability

The study data are considered commercially proprietary and are not stored for unrestricted access. All the authors have full access to the study data and take responsibility for the integrity of the data and the accuracy of the data analysis.

## Abbreviations

CT: Computerized tomography
GI: Gastrointestinal
GPC3: Glypican-3
HSP: Heat shock protein
ICB: Immune checkpoint blockade
MRI: Magnetic resonance imaging
TIGIT: T cell immunoreceptor with immunoglobulin and ITIM domains
TIM-3: T cell immunoglobulin and mucin-domain containing-3
ULN: Upper limit of normal
