# Supplementary material for: A phase I study of multi-HLA-binding peptides derived from heat shock protein 70/glypican-3 and a novel combination adjuvant of hLAG-3Ig and Poly-ICLC for patients with metastatic gastrointestinal cancers: YNP01 trial
Source: Cancer Immunol Immunother. 2020 Mar 26;69(8):1651–62. doi: 10.1007/s00262-020-02518-7 (PMC7347520; doi:10.1007/s00262-020-02518-7)
Supplement: Supplementary file 1 — Supplementary material 1 (PDF 57 kb) [file 262_2020_2518_MOESM1_ESM.pdf]

Supplementary table

Table S1. Candidate epitope peptides derived from GPC3

peptide	Position	Amino acid sequence	Affinities of each peptide for the HLAs		
			A*24:02	A*02:01	A*02:06
			log Kd	log Kd	log Kd
GPC-1	***166***	***MVNELFDSL***	***-4.69***	***-5.3***	***-6.14***
GPC-2	***170***	***LFDSLFPVI***	***-7.42***	***-5.07***	***> -3***
GPC-3	190	SALDINECL	> -3	> -3	-6.32
GPC-4	***222***	***SLQVTRIFL***	***-5.34***	***-6.21***	***-5.21***
GPC-5	407	GYICSHSPV	-6.55	> -3	> -3
GPC-6	232	ALNLGIEVI	-3.96	-5.92	-4.16
GPC-7	512	GMIKVKNQL	-6.9	-4.76	-3.36
GPC-8	85	ARLNMEQLL	-5.2	-3.84	-2.76

Table S2. Antigen-peptide reactive immune response

Level	Patient Number	HLA allele	Result of ELISPOT Assay (HSP70/GPC3): Pre-treatment	1 course	2 course	3 course
Level 1	1-1	24:02	- / 1+	NE	- / 2+	2+ / 2+
	1-2	24:02	1+ / 4+	2+ / 3+	4+ / 1+	
	1-3	24:02	1+ / -	1+ / 1+		
Level 2	2-1	02:06	NE	1+ / 1+	3+ / 3+	NE
	2-2	02:01	3+ / 5+	3+ / 1+	- / -	3+ / 2+
	2-3	24:02	1+ / 1+	1+ / 2+	1+ / 1+	1+ / 1+
Level 3	3-1	24:02	3+ / -	5+ / 4+	3+ / -	- / 1+
	3-2	02:06	1+ / 4+	3+ / -	1+ / 3+	1+ / -
		24:02	- / -	1+ / -	- / -	2+ / 1+
	3-3	02:01	3+ / 3+	3+ / 4+	1+ / 1+	
	3-4	24:02	1+ / 1+	3+ / 1+	3+ / 3+	
	3-5	02:01	1+ / 1+	1+ / 4+	4+ / 1+	
	3-6	24:02	1+ / 1+	2+ / 5+	5+ / 4+	1+ / 1+
	3-7	02:06	2+ / 4+	3+ / 3+	1+ / -	
		24:02	3+ / 4+	4+ / 4+	2+ / -	
	3-8	02:01	1+ / 1+	- / 1+		
	3-9	02:06	1+ / -	4+ / 1+	4+ / 1+	
		24:02	1+ / -	5+ / 2+	4+ / -	
	3-10	02:06	4+ / 1+	- / 1+	3+ / 4+	
		02:01	3+ / -	1+ / 4+	3+ / 3+	
	3-11	24:02	1+ / 1+	3+ / 2+	- / -	

ND: Not done, NE: Not evaluate

Table S3. All adverse events by maximum grade per patient

	Treatment Level (No. of patients)					
	Level 1 (n=3)		Level 2 (n=3)		Level 3 (n=11)	
	Grading of Adverse Events (CTCAE v4.0)					
Adverse event	G1/2	G3/4	G1/2	G3/4	G1/2	G3/4
Anemia	1		1		1	
Appendicitis				1		
Appetite loss				2		
Ascites		1				
Aspiration				1		
Back pain					1	
Bronchial stricture and hemorrhage				1		
Dysphagia						1
Dyspnea				1		
Edema	(1[a])				(1[a])	
Fever	1					
Gastrointestinal hemorrhage		1				
Hypercalcemia		1				
Hypertension						1
Hypoalbuminemia	1		1			
Hypokalemia		1				
Hyponatremia		1				
Ileus		1				1
Increased AST or ALT					1	1
Injection site reaction	(1[c])		(1[c])		(3[b,c])	
Intra-IVC tumor thrombus		1				
Nausea or vomiting		1	1			
Upper respiratory infection				1		
Tumor pain	1		1			

All adverse events, in parenthesis (*): related adverse events (a:possible, b:probable, c:definite), AST : Aspartate aminotransferase, ALT: Alanine aminotransferase
