# Supplementary material for: A phase I study of multi-HLA-binding peptides derived from heat shock protein 70/glypican-3 and a novel combination adjuvant of hLAG-3Ig and Poly-ICLC for patients with metastatic gastrointestinal cancers: YNP01 trial
Source: Cancer Immunol Immunother. 2020 Mar 26;69(8):1651–62. doi: 10.1007/s00262-020-02518-7 (PMC7347520; doi:10.1007/s00262-020-02518-7)
Supplement: Supplementary file 2 — Supplementary material 2 (DOCX 19 kb) [file 262_2020_2518_MOESM2_ESM.docx]

Table S4. Relationship between immunological factors and clinical outcomes

|  | Antigen expression  (Both HSP70 and GPC3)  (N=14) | | | CTL response  (HSP70 or GPC3)  (N=17) | | | TIM3/CD4 reduction after one course of vaccination  (N=17) | | |
| --- | --- | --- | --- | --- | --- | --- | --- | --- | --- |
|  | + | - | P value | + | - | P  value | + | - | P  value |
| Median survival time  (months) | 10.5 | 6.5 | 0.09 | 9.5 | Not  detected | 0.23 | 10.5 | 6.5 | 0.25 |
| Tumor marker  reduction | 2/4  (50%) | 6/10  (60%) | 0.73 | 11/15  (73%) | 0/2  (0%) | 0.03 | 9/13 (69%) | 2/4 (50%) | 0.49 |
