# Supplementary material for: A phase I study of multi-HLA-binding peptides derived from heat shock protein 70/glypican-3 and a novel combination adjuvant of hLAG-3Ig and Poly-ICLC for patients with metastatic gastrointestinal cancers: YNP01 trial
Source: Cancer Immunol Immunother. 2020 Mar 26;69(8):1651–62. doi: 10.1007/s00262-020-02518-7 (PMC7347520; doi:10.1007/s00262-020-02518-7)
Supplement: Supplementary file 3 — Supplementary material 3 (PDF 757 kb) [file 262_2020_2518_MOESM3_ESM.pdf]

Supplementary Figure

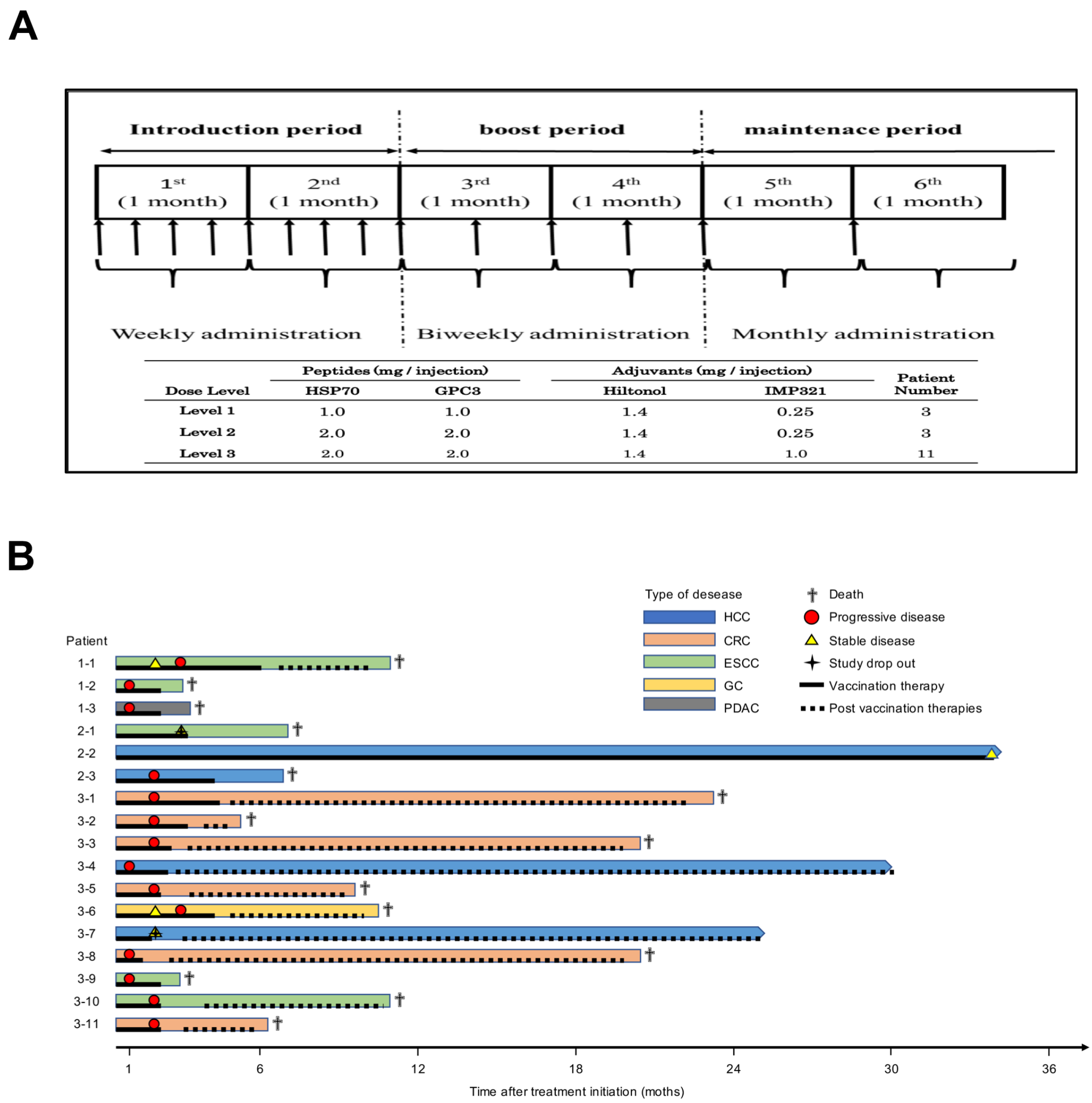

Fig. S1. Study design and clinical outcomes of enrolled patients.

A, Treatment schedule, dose escalation and cohort assignment of this study. B, Swimmer plot for enrolled patients (n = 17).

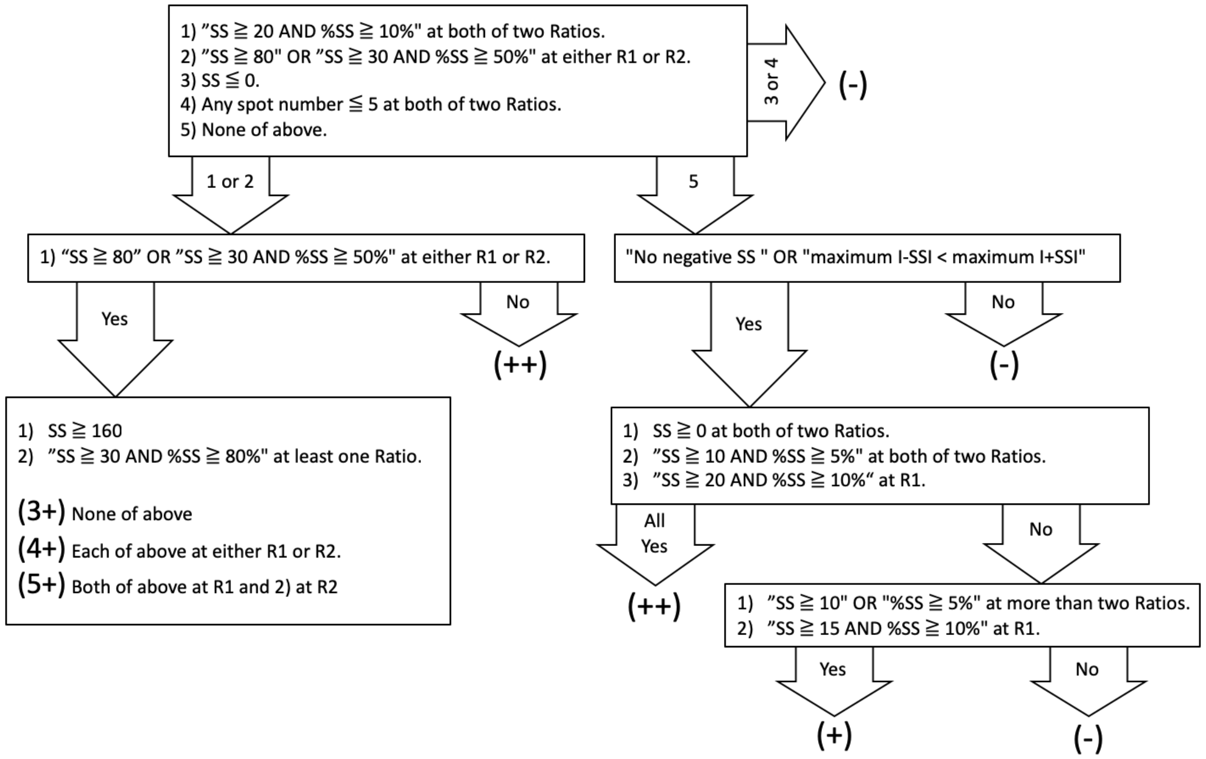

Fig. S2. ELISPOT assay protocol used in this study.

Positivity of antigen-specific T cell response was quantitatively defined according a tree algorithm. In brief, the peptide-specific spots (SS) were the averages of triplicates and were determined by subtracting the value of the HIV peptide-pulsed stimulator well from that of the immunized peptide-pulsed stimulator well. %SS is the percentage of SS among the average number of spots in the immunized peptide pulsed stimulator well. Positivity of antigen-specific T cell response was classified as −, +, 2+, 3+, 4+, or 5+ depending on the numbers of peptide-specific spots and the invariability of the peptide-specific spots at different responder/stimulator ratios. SS, peptide-specific spots; R1, responder/stimulator ratio = 1; R2, responder/stimulator ratio = 0.5.

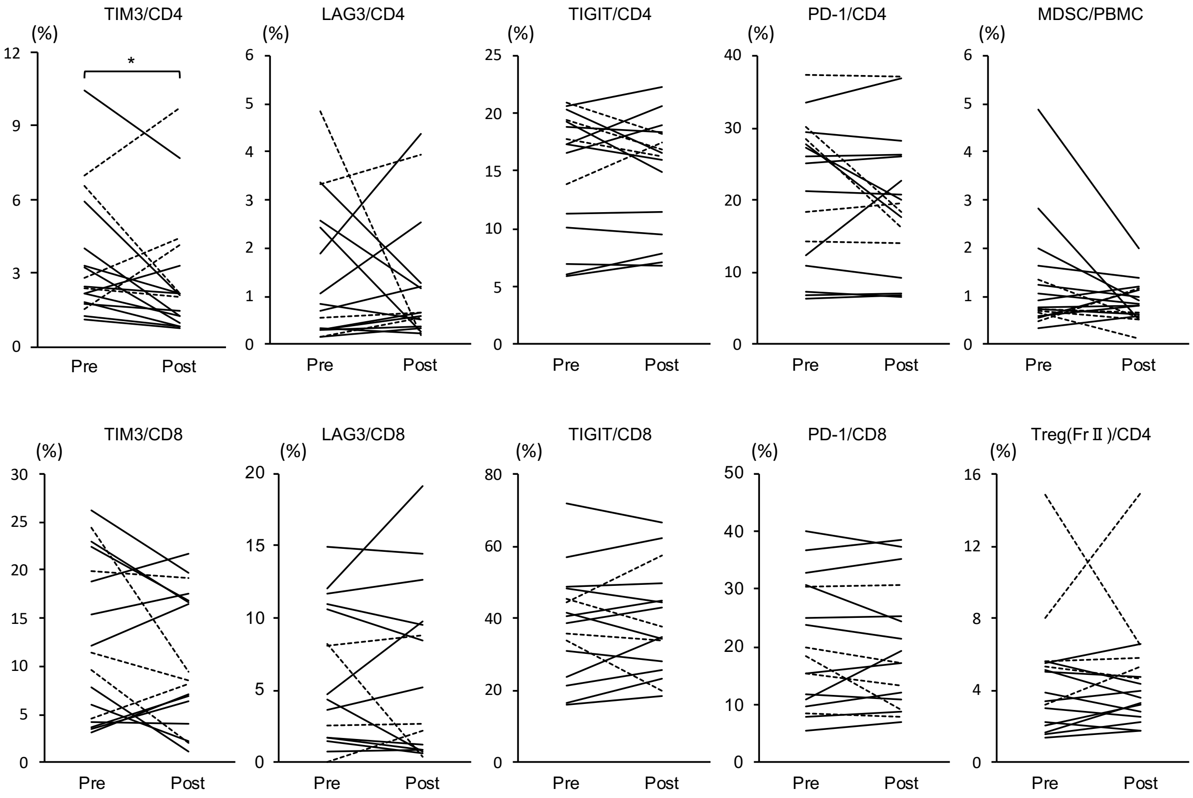

Fig. S3. Flow cytometlic analysis of surface marker expression on PBMCs before and after treatment

Comparison of the exhaustion marker expression levels in CD4 and CD8 T and immunosuppressive (Treg and MDSC) cells in the PBMCs before and after one course of treatment. (*, $P < 0.05$)
